# Non-contiguous finished genome sequence and description of *Corynebacterium jeddahense* sp. nov.

**DOI:** 10.4056/sigs.5561028

**Published:** 2014-04-29

**Authors:** Sophie Edouard, Fehmida Bibi, Ramasamy Dhamodharan, Jean-Christophe Lagier, Esam Ibraheen Azhar, Catherine Robert, Aurelia Caputo, Muhammad Yasir, Asif Ahmad Jiman-Fatani, Maha Alawi, Pierre-Edouard Fournier, Didier Raoult

**Affiliations:** 1 Unité de Recherche sur les Maladies Infectieuses et Tropicales Emergentes, Institut Hospitalo-Universitaire Méditerranée-Infection, Faculté de médecine, Aix-Marseille Université, Boulevard Jean Moulin, Marseille cedex, France; 2 Special Infectious Agents Unit, King Fahd Medical Research Center, King Abdul Aziz University, Jeddah, Saudi Arabia.; 3 Medical Laboratory Technology Department, Faculty of Applied Medical Sciences, King Abdulaziz University, Jeddah, Saudi Arabia.; 4 Department of Medical Microbiology and Parasitology, Faculty of Medicine, King Abdulaziz University, Jeddah, Saudi Arabia; 5 Infection Control Unit, King Abdulaziz University Hospital, King Abdulaziz University, Jeddah, Saudi Arabia

**Keywords:** *Corynebacterium jeddahense*, genome, culturomics, taxono-genomics

## Abstract

*Corynebacterium jeddahense* sp. nov., strain JCB^T^, is the type strain of *Corynebacterium jeddahense* sp. nov., a new species within the genus *Corynebacterium*. This strain, whose genome is described here, was isolated from fecal flora of a 24-year-old Saudi male suffering from morbid obesity. *Corynebacterium jeddahense* is a Gram-positive, facultative anaerobic, nonsporulating bacillus**.** Here, we describe the features of this bacterium, together with the complete genome sequencing and annotation, and compare it to other member of the genus *Corynebacterium.* The 2,472,125 bp-long genome (1 chromosome but not plasmid) contains 2,359 protein-coding and 53 RNA genes, including 1 rRNA operon.

## Introduction

*Corynebacterium jeddahense* strain JCB^T^ (= CSUR P778 = DSM 45997) is the type strain of *C. jeddahense* sp. nov. This bacterium is a Gram-positive bacillus, non-spore-forming, strictly aerobic and non-motile that was isolated from the feces of a 24 year-old man living in Jeddah, Saudi Arabia, who suffered from morbid obesity. This isolation was part of a “culturomics” study aiming at cultivating the maximum number of bacterial species from human feces [1,2].

The current classification of bacteria remains a matter of debate and relies on a combination of phenotypic and genomic characteristics [3]. Currently, more than 12,000 bacterial genomes have been sequenced [4], and we recently proposed an innovative concept for the taxonomic description of new bacterial species that integrates their genomic characteristics [5-35] as well as proteomic information obtained by MALDI-TOF-MS analysis [36].

In the present study, we present a summary classification and a set of features for *Corynebacterium jeddahense* sp. nov., strain JCB^T^ (CSUR P778 = DSM 45997), including the description of its complete genome sequence and annotation. These characteristics support the circumscription of the species *Corynebacterium jeddahense.* The genus *Corynebacterium* was created in 1896 by Lehmann and Neumann and currently consists of mainly Gram-positive, non-spore-forming, rod-shaped bacteria with a high DNA G+C content [37]. This genus belongs to the phylum *Actinobacteria* and currently includes more than 100 species with standing in nomenclature [38]. Members of the genus *Corynebacterium* are found in various environments including water, soil, sewage, and plants as well as in human normal skin flora and human or animals clinical samples. Some *Corynebacterium* species are well-established human pathogens while others are only considered as opportunistic pathogens. *Corynebacterium diphteriae*, causing diphtheria, is the most significant pathogen in this genus [39]. However, many *Corynebacterium* species including, among others, *C. jeikeium*, *C. urealyticum*, *C. striatum, C. ulcerans* and *C. pseudotuberculosis*, are recognized agents of bacteremias, endocarditis, urinary tract infections, and respiratory or wound infections [40].

## Classification and features

A stool sample was collected from a 24-year-old man living in Jeddah, Saudi Arabia, who suffered from morbid obesity (BMI=52). The patient gave a signed informed consent. The study and the assent procedure were approved by the Ethics Committees of the King Abdulaziz University, King Fahd medical Research Center, Saudi Arabia, under agreement number 014-CEGMR-2-ETH-P, and of the Institut Fédératif de Recherche 48, Faculty of Medicine, Marseille, France, under agreement number 09-022. The patient was not taking any antibiotics at the time of stool sample collection and the fecal sample was kept at -80°C after collection. Strain JCB^T^ (Table 1) was first isolated in July 2013 by cultivation on 5% sheep blood-enriched Columbia agar (BioMerieux, Marcy l’Etoile, France) in aerobic atmosphere with 5% CO_2_ at 37°C after a 14-day preincubation of the stool sample in an aerobic blood culture bottle that also contained sterile rumen sheep fluid. Several other new bacterial species were isolated from this stool specimen using various culture conditions.

**Table 1:** Classification and general features of *C. jeddahense* strain JCB^T^ according to the MIGS recommendations [41].

MIGS ID	Property	Term	Evidence code^a^
		Domain *Bacteria*	TAS [42]
		Phylum *Actinobacteria*	TAS [43]
		Class *Actinobacteria*	TAS [44]
	Current classification	Order *Actinomycetales*	TAS [44-47]
		Family *Corynebacteriaceae*	TAS [44-46,48]
		Genus *Corynebacterium*	TAS [45,49,50]
		Species *Corynebacterium jeddahense*	IDA
		Type strain JCB^T^	IDA
	Gram stain	Positive	IDA
	Cell shape	Rod	IDA
	Motility	not motile	IDA
	Sporulation	Non sporulating	IDA
	Temperature range	Mesophilic	IDA
	Optimum temperature	37°C	IDA
MIGS-6.3	Salinity	Unknown	IDA
MIGS-22	Oxygen requirement	Aerobic	IDA
	Carbon source	Unknown	NAS
	Energy source	Unknown	NAS
MIGS-6	Habitat	Human gut	IDA
MIGS-15	Biotic relationship	Free living	IDA
MIGS-14	Pathogenicity	Unknown	
	Biosafety level	2	
	Isolation	Human feces	
MIGS-4	Geographic location	Jeddah, Saudi Arabia	IDA
MIGS-5	Sample collection time	July 2013	IDA
MIGS-4.1	Latitude Longitude	21.422487 39.826184	IDA
MIGS-4.3	Depth	Surface	IDA
MIGS-4.4	Altitude	0 m above sea level	IDA

^a^Evidence codes - IDA: Inferred from Direct Assay; TAS: Traceable Author Statement (i.e., a direct report exists in the literature); NAS: Non-traceable Author Statement (i.e., not directly observed for the living, isolated sample, but based on a generally accepted property for the species, or anecdotal evidence). These evidence codes are from the Gene Ontology project [51]. If the evidence is IDA, then the property was directly observed for a live isolate by one of the authors or an expert mentioned in the acknowledgements.

**Figure 1:**
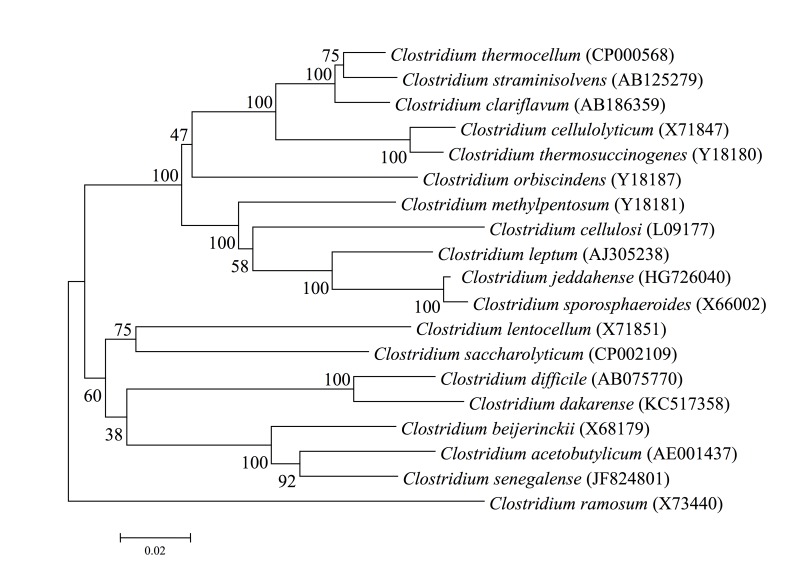
Phylogenetic tree highlighting the position of *Corynebacterium jeddahense* strain JCB^T^ relative to other type strains within the genus *Corynebacterium*. GenBank accession numbers are indicated in parentheses. Sequences were aligned using CLUSTALW, and phylogenetic inferences obtained using the maximum-likelihood method in the MEGA software package. Numbers at the nodes are percentages of bootstrap values obtained by repeating the analysis 500 times to generate a majority consensus tree. *Mycobacterium avium* was used as outgroup. The scale bar represents 1% nucleotide sequence divergence.

This strain exhibited a 96.8% nucleotide sequence similarity with *C. coyleae*, the phylogenetically most closely related *Corynebacterium* species with a validly published name (Figure 1). The similarity value was lower than the 98.7% 16S rRNA gene sequence threshold recommended by Stackebrandt and Ebers to delineate a new species without carrying out DNA-DNA hybridization [52], and was in the 82.9 to 99.60% range observed among members of the genus *Corynebacterium* with standing in the nomenclature [53].

**Figure 2:**
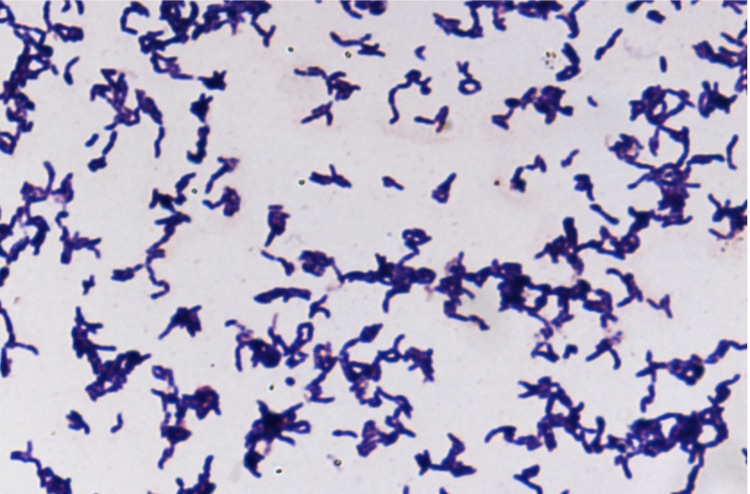
Gram-stain of *C. jeddahense* strain JCB^T^

**Figure 3:**
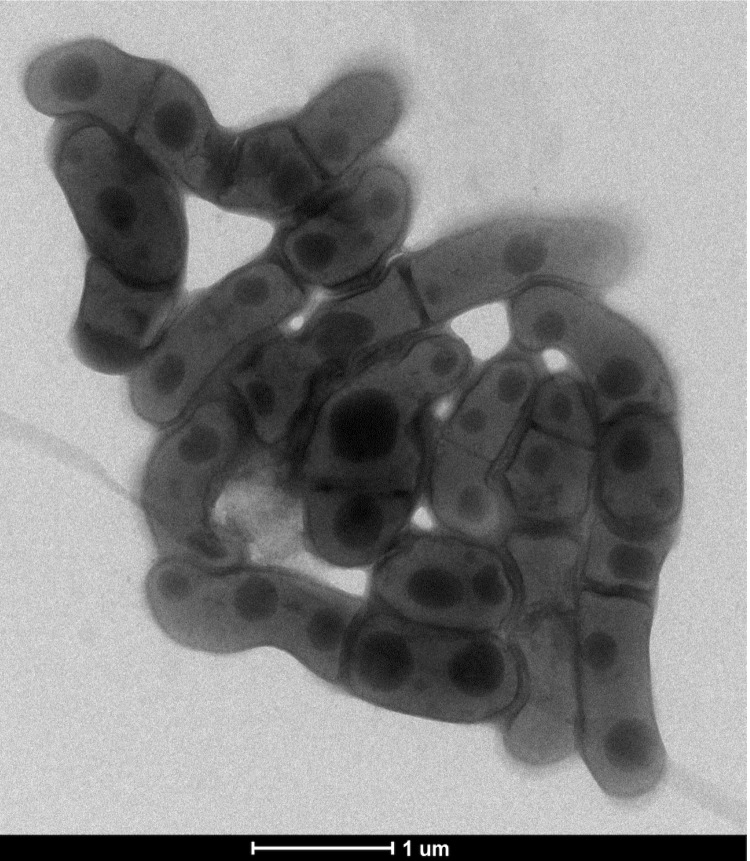
Transmission electron micrograph of *C. jeddahense* strain JCB^T^, taken using a Morgani 268D (Philips) at an operating voltage of 60kV. The scale bar represents 1 µm.

Four growth temperatures (25, 30, 37, 45°C) were tested. Growth occurred between 30 and 45°C on blood-enriched Columbia agar (BioMerieux), with the optimal growth being obtained at 37°C after 48 hours of incubation. Growth of the strain was tested under anaerobic and microaerophilic conditions using GENbag Anaer and GENbag microaer systems, respectively (BioMerieux), and under aerobic conditions, with or without 5% CO_2_. Optimal growth was achieved aerobically. Weak cell growth was observed under microaerophilic and anaerobic conditions. The motility test was negative and the cells were not sporulating. Colonies were translucent and 1 mm in diameter on blood-enriched Columbia agar. Cells were Gram-positive rods (Figure 2). In electron microscopy, the bacteria grown on agar had a mean diameter and length of 0.63 and 1.22 μm, respectively (Figure 3).

Strain JCB^T^ was catalase positive and oxidase negative. Using an API CORYNE strip, a positive reaction was observed only for alkaline phosphatase and for catalase. Negative reactions were observed for reduction of nitrates, pyrolidonyl arylamidase, pyrazinamidase, β-glucuronidase, β-galactosidase, α-glucosidase N-acetyl-β-glucosaminidase, β-glucosidase, urease, gelatin hydrolysis and fermentation of glucose, ribose xylose, mannitol, maltose, lactose, saccharose and glycogen. Using the Api Zym system (BioMerieux), alkaline and acid phosphatases and Naphtol-AS-BI phosphohydrolase activities were positive, but esterase (C4), esterase lipase (C8), lipase (C14), trypsin, α-chemotrypsin, α-galactosidase, β-galactosidase, β-glucuronidase, α-glucosidase, N actetyl-β-glucosaminidase, leucine arylamidase, valine arylamidase, cystin arylamidase, α-mannosidase and α-fucosidase activities were negative.

Substrate oxidation and assimilation were examined with an API 50CH strip (BioMerieux) at 37°C. All reactions were negative, including fermentation of starch, glycogen, glycerol, erythritol, esculin ferric citrate, amygdalin, arbutin, salicin, L-arabinose, D-ribose, D-xylose, methyl β-D-xylopyranoside, D-galactose, D-glucose, D-fructose, D-mannose, L-rhamnose, D-mannitol, methyl α-D-xylopyranoside, methyl α-D-glucopyranoside, N-acetylglucosamine, D-cellobiose, D-maltose, D-lactose, D-melibiose, D-saccharose, D-trehalose, inulin, D-raffinose, D-lyxose, D-arabinose, L-xylose, D-adonitol, L-sorbose, dulcitol, inositol, D-sorbitol, D-melezitose, D-xylitol, gentiobiose, D-turanose, D-tagatose, D-fucose, L-fucose, D-arabitol, L-arabitol, potassium gluconate, and potassium 2-ketogluconate.

*C. jeddahense* is susceptible to amoxicillin, ceftriaxone, imipenem, rifampin, gentamicin, doxycycline and vancomycin, but resistant to ciprofloxacin, trimethoprim/sulfamethoxazole, eyrthromycin and metronidazole. When compared with representative species from the genus *Corynebacterium*, *C. jeddahense* strain JCB^T^ exhibited the phenotypic differences detailed in Table 2.

**Table 2:** Differential characteristics of *C. jeddahense* strain JCB^T^ and closely related strains.

	*C. jeddahense*	*C. pseudotuberculosis*	*C. efficiens*	*C. glutamicum*	*C. lipophiloflavum*	*C. coyleae*	*C. glaucum*
Diameter x length (µm)	0.63 x 1.22	0.5-0.6 x 1.0-3.3	0.8-1.1 x 1.0-4.5	0.7-1 x 1-3	1-3	NA	NA
Oxygen requirement	Aero-anaerobic	Aero-anaerobic	Aero-anaerobic	Aero-anaerobic	Aero-anaerobic	Aero-anaerobic	Aero-anaerobic
Pigment production	None	Yellowish-white	Yellow	Pale yellow to yellow	Yellow	None	Light grey
Gram stain	+	+	+	+	+	+	+
Motility	-	-	-	-	-	-	-
Endospore formation	-	-	-	-	-	-	-
**Production of**							
Acid phosphatase	+	NA	NA	-	+	+	-
Alkaline phosphatase	+	v	-	-	+	+	+
Catalase	+	+	+	+	+	+	+
Oxidase	-	-	-	-	-	-	-
Pyrazinamidase	-	-	+	-	+	+	+
Nitrate reductase	-	V	+	+	-	-	-
Urease	-	+	V	+	W	-	-
**Utilization of**							
Ribose	-	+	+	-	-	+	-
Mannose	-	+	+	+	NA	-	-
Mannitol	-	-	-		-	+	-
Sucrose	-	+		+	-	-	+
D-glucose	-	+	+	+	-	+	+
D-fructose	-	+	+	+	NA	+	NA
D-maltose	-	+	+	+	-	+	-
D-lactose	-	-	-	-	-	-	-
**Habitat**	Human gut	Sheep, infected gland, South America	Soil, Japan	Sewage, Japan	vaginal swab, Switzerland	Human blood	Cosmetic dye
Optimal temperature (^o^C)	37°C	37°C	30-40°C	25-37°C	37°C	37°C	37°C

+, Positive; –, negative; V, variable, W, weak reaction; NA, not available.

**Figure 4:**
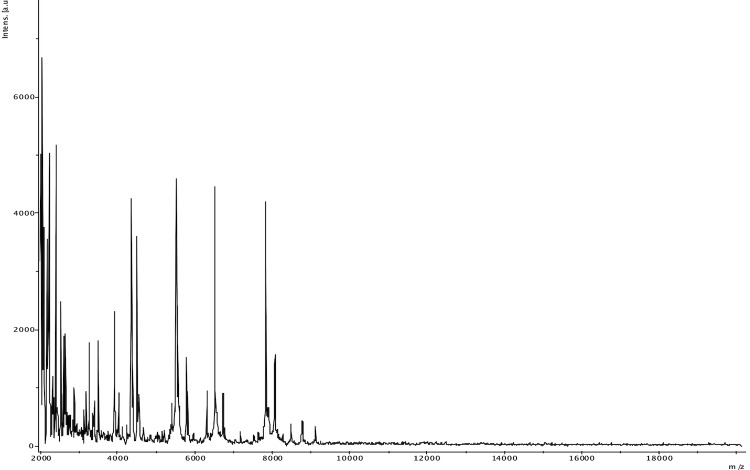
Reference mass spectrum from *C. jeddahense* strain JCB^T^. Spectra from 12 individual colonies were compared and a reference spectrum was generated.

**Figure 5:**
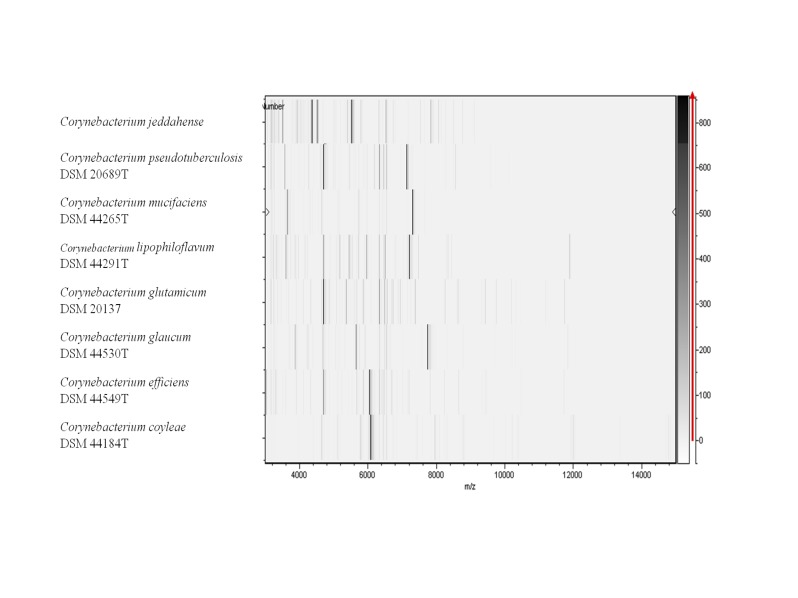
Gel view comparing *C. jeddahense* strain JCB^T^ (= CSUR P778 = DSM 45997) to other species from the genus *Corynebacterium*. The gel view displays the raw spectra of loaded spectrum files as a pseudo-electrophoretic gel. The x-axis records the m/z value. The left y-axis displays the running spectrum number originating from subsequent spectra loading. The peak intensity is expressed by a grey scale scheme code. The grey scale bar on the right y-axis indicates the relation between the shade of grey of the “band” and the peak intensity, in arbitrary units. Displayed species are indicated on the left.

*C. pseudotuberculosis* strain CIP 102968^T^ [54], *C. efficiens* YS-314^T^ [55], *C. glutamicum* strain ATCC 13032^T^ [56], *C. lipophiloflavum* strain DSM 44291^T^ [57], *C. coyleae* strain DSM44184^T^ [58] and *C. glaucum* strain IMMIB R-5091^T^ [59].

t (MALDI-TOF) MS protein analysis was carried out as previously described [36] using a Microflex spectrometer (Brüker Daltonics, Leipzig, Germany). Twelve individual colonies were deposited on a MTP 384 MALDI-TOF target plate (Brüker). The twelve spectra were imported into the MALDI BioTyper software (version 2.0, Brüker) and analyzed by standard pattern matching (with default parameter settings) against the main spectra of 4,706 bacteria, including 169 spectra from 69 validly named *Corynebacterium* species used as reference data in the BioTyper database. The score generated enabled the presumptive identification and discrimination of the tested species from those in a database: a score > 2 with a validated species enabled the identification at the species level; and a score < 1.7 did not enable any identification. For strain JCB^T^, no significant score was obtained, suggesting that our isolate was not a member of any known species (Figures 4 and 5).

## Genome sequencing information

### Genome project history

The organism was selected for sequencing on the basis of its phylogenetic position, 16S rDNA similarity and phenotypic differences with members of the genus *Corynebacterium* and is part of a culturomics study of the human digestive flora aiming at isolating all bacterial species within human feces [2]. It was the 96th genome from a *Corynebacterium* species. The EMBL accession number is CBYN00000000and consists of 244 contigs. Table 3 shows the project information and its association with MIGS version 2.0 compliance [41].

**Table 3:** Project information

**MIGS ID**	**Property**	**Term**
MIGS-31	Finishing quality	High-quality draft
MIGS-28	Libraries used	One paired-end 454 3-kb library
MIGS-29	Sequencing platforms	454 GS FLX Titanium
MIGS-31.2	Fold coverage	130
MIGS-30	Assemblers	Newbler version 2.5.3
MIGS-32	Gene calling method	Prodigal
	BioProject ID	PRJEB4941
	GenBank Accession number	CBYN00000000
	GenBank date of release	February 12, 2014
MIGS-13	Project relevance	Study of the human gut microbiome

### Growth conditions and DNA isolation

*C. jeddahense* sp. nov strain JCB^T^ (= CSUR P778 = DSM 45997) was grown aerobically on sheep blood-enriched Columbia agar medium at 37°C. Two petri dishes were spread and resuspended in 6x100µl of G2 buffer (EZI DNA Tissue Kit, Qiagen). A first mechanical lysis was performed using glass powder on the Fastprep-24 device (Sample Preparation System, MP Biomedicals, USA) using 2x20 second bursts. DNA was treated with 2.5µg/µL of lysozyme for 30 minutes at 37°C) and extracted using the BioRobot EZ 1 Advanced XL (Qiagen).The DNA was then concentrated and purified on a Qiamp kit (Qiagen). The DNA concentration, as measured by the Qubit assay with the high sensitivity kit (Life Technologies, Carlsbad, CA, USA), was 3.1ng/µl.

### Genome sequencing and assembly

Genomic DNA of *C. jeddahense* was sequenced on a MiSeq sequencer (Illumina Inc, San Diego, CA, USA) using both paired-end and mate-pair sequencing with the Nextera XT DNA sample and Nextera Mate Pair sample prep kits, respectively (Illumina).

To prepare the paired-end library, Genomic DNA was diluted 1:3 to obtain a 1ng/µl concentration. The “tagmentation” step fragmented and tagged the DNA with a mean size of 1.4kb. Then, a limited PCR amplification (12 cycles) completed the tag adapters and introduced dual-index barcodes. After purification on AMPure XP beads (Beckman Coulter Inc, Fullerton, CA, USA), the library was then normalized on specific beads according to the Nextera XT protocol (Illumina). The pooled single strand library was loaded onto the reagent cartridge and then onto the instrument along with the flow cell. Automated cluster generation and paired end sequencing with dual index reads were performed in a single 39-hours run in 2x250-bp. Total information of 5.3Gb was obtained from a 574 K/mm^2^ cluster density with a cluster passing quality control filters of 95.4% (11,188,000 clusters). Within this run, the index representation for *Corynebacterium jeddahense* was determined to 6.2%. The 641,099 reads were filtered according to the read qualities.

The mate-pair library was prepared with 1µg of genomic DNA using the Nextera mate-pair Illumina guide. The genomic DNA sample was simultaneously fragmented and tagged with a mate-pair junction adapter. The profile of the fragmentation was validated on an Agilent 2100 BioAnalyzer (Agilent Technologies Inc, Santa Clara, CA, USA) with a DNA 7500 labchip. The DNA fragments ranged in size from 1kb up to 10kb with a mean size of 2.6kb. No size selection was performed and 105ng of tagmented fragments were circularized. The circularized DNA was mechanically sheared to small fragments with an optimal at 409bp on the Covaris device S2 in microtubes (Covaris, Woburn, MA, USA).The library profile was visualized on a High Sensitivity Bioanalyzer LabChip (Agilent Technologies Inc, Santa Clara, CA, USA). After a denaturation step and dilution at 10pM, the library was loaded onto the reagent cartridge and then onto the instrument along with the flow cell. Automated cluster generation and sequencing run were performed in a single 42-hour run in a 2x250-bp. Total information of 3.9Gb was obtained from a 399 K/mm^2^ cluster density with a cluster passing quality control filters of 97.9% (7,840,000 clusters). Within this run, the index representation for *Corynebacterium jeddahense* was determined to 8.17%. The 626,585 reads were filtered according to the read qualities. Genome assembly was performed using Newbler (Roche).

### Genome annotation

Open Reading Frames (ORFs) were predicted using Prodigal [60] with default parameters. However, the predicted ORFs were excluded if they spanned a sequencing gap region. The predicted bacterial protein sequences were searched against GenBank [61] and Clusters of Orthologous Groups (COG) databases using BLASTP. The tRNAs and rRNAs were predicted using the tRNAScanSE [62] and RNAmmer [63] tools, respectively. Lipoprotein signal peptides and numbers of transmembrane helices were predicted using SignalP [64] and TMHMM [65], respectively. ORFans were identified if their BLASTP E-value was lower than 1e^-3^ for alignment length greater than 80 amino acids. If alignment lengths were smaller than 80 amino acids, we use an *E*-value of 1e^-5^. Such parameter thresholds have already been used in previous works to define ORFans.

Artemis [66] and DNA Plotter [67] were used for data management and visualization of genomic features, respectively. The Mauve alignment tool (version 2.3.1) was used for multiple genomic sequence alignments [68]. To estimate the mean level of nucleotide sequence similarity at the genome level between *C. jeddahense* and another 4 members of the *Corynebacterium* genus (Tables 6A and 6B), we used the Average Genomic Identity Of gene Sequences (AGIOS) home-made software [35]. Briefly, this software combines the Proteinortho software [69] for detecting orthologous proteins between genomes compared two by two, then retrieves the corresponding genes and determines the mean percentage of nucleotide sequence identity among orthologous ORFs using the Needleman-Wunsch global alignment algorithm.

**Table 6:** Genomic comparison of *C. jeddahense* and 4 other *Corynebacterium* species. *^†^*

**Species**	**Strain**	**Genome accession number**	**Genome size (Mb)**	**G+C content**
*C. jeddahense*	JCB^T^	CBYN00000000	2,472,125	67.2
*C. efficiens*	YS-314^T^	NC_004369	3,147,090	62.9
*C. lipophiloflavum*	DSM 44291^T^	ACHJ00000000	2,293,743	64.8
*C. glutamicum*	ATCC 13032^T^	NC_003450	3,309,401	53.8
*C. pseudotuberculosis*	CIP 52.97	NC_017307	2,320,595	52.1

*^†^*Species name, strain, GenBank accession number, genome size and G+C content of compared genomes.

## Genome properties

**Figure 6:**
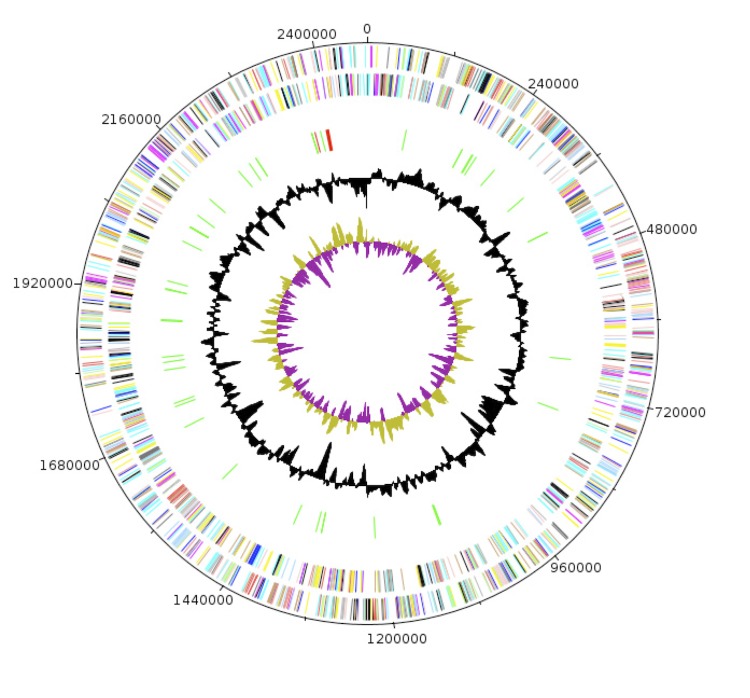
Graphical circular map of the *C. jeddahense* strain JCB^T^ genome. From the outside in, the outer two circles shows open reading frames oriented in the forward (colored by COG categories) and reverse (colored by COG categories) directions, respectively. The third circle marks the rRNA gene operon (red) and tRNA genes (green). The fourth circle shows the G+C% content plot. The inner-most circle shows GC skew, purple indicating negative values whereas olive for positive values.

The genome *C. jeddahense* strain JCB^T^ is 2,472,125 bp long (one chromosome, no plasmid) with a G+C content of 67.2% (Figure 6, Table 4). Of the 2,412 predicted chromosomal genes, 2,359 were protein-coding genes and 53 were RNAs. A total of 1,462 genes (60.61%) were assigned a putative function. Sixty-seven genes were identified as ORFans (2.77%) and the remaining genes were annotated as hypothetical proteins. The properties and statistics of the genome are summarized in Table 4. The distribution of genes into COGs functional categories is presented in Table 5.

**Table 4:** Nucleotide content and gene count levels of the Chromosome

**Attribute**	Value	% of total^a^
Genome size (bp)	2,472,125	
DNA G+C content (bp)	1,661,268	67.2
DNA coding region (bp)	2,235,018	87.17
Extrachromosomal elements	0	
Total genes	2,412	100
RNA genes	53	2.2
Protein-coding genes	2,359	97.8
Genes with function prediction	1,462	60.61
Genes assigned to COGs	1,636	67.82
Genes with peptide signals	187	7.75
Genes with transmembrane helices	629	26.1

^a^ The total is based on either the size of the genome in base pairs or the total number of protein coding genes in the annotated genome

**Table 5:** Number of genes associated with the 25 general COG functional categories

**Code**	**Value**	**% age**^a^	**Description**
J	149	6.18	Translation
A	1	0.04	RNA processing and modification
K	132	5.47	Transcription
L	154	6.38	Replication, recombination and repair
B	0	0	Chromatin structure and dynamics
D	22	0.91	Cell cycle control, mitosis and meiosis
Y	0	0	Nuclear structure
V	32	1.32	Defense mechanisms
T	57	2.36	Signal transduction mechanisms
M	104	4.31	Cell wall/membrane biogenesis
N	1	0.04	Cell motility
Z	0	0	Cytoskeleton
W	0	0	Extracellular structures
U	21	0.87	Intracellular trafficking and secretion
O	58	2.2	Posttranslational modification, protein turnover, chaperones
C	85	3.52	Energy production and conversion
G	109	4.52	Carbohydrate transport and metabolism
E	191	7.1	Amino acid transport and metabolism
F	66	2.73	Nucleotide transport and metabolism
H	85	3.52	Coenzyme transport and metabolism
I	47	1.95	Lipid transport and metabolism
P	135	5.6	Inorganic ion transport and metabolism
Q	40	1.66	Secondary metabolites biosynthesis, transport and catabolism
R	232	9.62	General function prediction only
S	145	6.01	Function unknown
-	776	32.17	Not in COGs

^a^ The total is based on the total number of protein coding genes in the annotated genome

## Genome comparison of C. jeddahense** with 4 other *Corynebacterium* genomes

**Figure 7:**
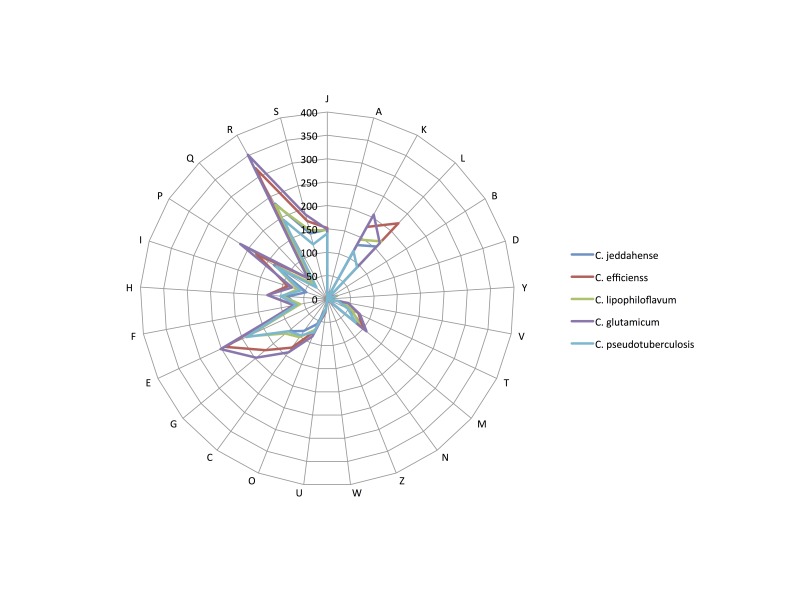
Distribution of functional classes of predicted genes in the genomes from *C. jeddahense* JCB^T^ (colored in sea blue), *C. efficiens* YS-314^T^ (blue), *C. lipophiloflavum* strain DSM 44291^T^ (green), *C. glutamicum* strain ATCC 13032^T^ (yellow) and *C. pseudotuberculosis* strain CIP 102968^T^ (red) chromosomes, according to the clusters of orthologous groups of proteins.

We compared the genome of C. jeddahense** strain JCB^T^ with those of *C. efficiens* YS-314^T^, *C. lipophiloflavum* strain DSM 44291^T^, *C. glutamicum* strain ATCC 13032^T^ and *C. pseudotuberculosis* strain CIP 102968^T^ (Table 6 and 7). The draft genome sequence of *C. jeddahiense* strain JCB^T^ is larger than those of *C. efficiens, C. lipophiloflavum* and *C. glutamicum* (2.47, 2.26, 2.43 and 2.11 Mb, respectively), but smaller than that of *C. pseudotuberculosis* (2.48 Mb). The G+C content of *C. jeddahense* is larger than those of C. efficiens*, C. lipophiloflavum*, *C. glutamicum* and *C. pseudotuberculosis* (67.2, 62.9, 64.8, 53.8, and 52.1%, respectively). The gene content of *C. jeddahense* (2,359) is smaller than those of *C. efficiens, C. lipophiloflavum* and *C. glutamicum* (2,398, 2,371 and 2,993, respectively) but larger that of *C. pseudotuberculosis* (2,060). The distribution of genes into COG categories was similar but not identical in all four compared genomes (Figure 7).

**Table 7:** Genomic comparison of C. jeddahense and 4 other Corynebacterium species. ^†^

	*C. jeddahense*	*C. efficiens*	*C. lipophiloflavum*	*C. glutamicum*	*C. pseudotuberculosis*
*C. jeddahense*	**2359**	1,369	1,345	1,385	1,230
*C. efficiens*	71.81	**2,938**	1,449	1,605	1,381
*C. lipophiloflavum*	77.26	71.34	**2,2371**	1,465	1,285
*C. glutamicum*	68.12	75.04	68.43	**2,993**	1,400
*C. pseudotuberculosis*	66.44	67.93	66.7	68.47	**2,060**

*^†^*Numbers of orthologous proteins shared between genomes (upper right); AGIOS values (lower left); numbers of proteins per genome (bold).

In addition, *C. jeddahense* shared 1,369, 1,345, 1,385 and 1,230 orthologous genes with *C. efficiens*, *C. lipophiloflavum, C. glutamicum* and *C. pseudotuberculosis*, respectively. The AGIOS value ranged from 66.7 to 75.04 among compared *Corynebacterium* species except *C. jeddahense*. When compared to other species, the AGIOS value ranged from 66.44% with *C. pseudotuberculosis* to 77.26% with *C. lipoflavum*, thus confirming its new species status (Table 6B).

## Conclusion

On the basis of phenotypic, phylogenetic and genomic analyses, we formally propose the creation of *Corynebacterium jeddahense* sp. nov., that contains the strain JCB^T^. The strain has been isolated from the fecal flora of a Saudi man suffering from morbid obesity. Several other as yet undescribed bacterial species were also cultivated from different fecal samples through diversification of culture conditions [5-35], thus suggesting that the human fecal flora of humans remains partially unknown.

### Description of *Corynebacterium jeddahense* sp. nov.

*Corynebacterium jeddahense* (jed.dah.en'se N.L. neut. adj. Jeddah the name of the town in Saudi Arabia where the specimen was obtained).

Grows occurred between 30 and 45°C on blood-enriched Columbia agar (BioMerieux). Optimal growth obtained at 37°C in aerobic atmosphere. Weak growth obtained in microaerophilic and anaerobic conditions. Colonies are translucent and 1 mm in diameter. Not motile, not endospore-forming. Cells are Gram-positive rods and have a mean diameter and length of 0.63 and 1.22 μm, respectively. Catalase positive, oxidase negative. Using the API Coryne (BioMerieux) system, cells are alkaline phosphatase positive but negative for reduction of nitrates, pyrolidonyl arylamidase, pyrazinamidase, β-glucuronidase, β-galactosidase, α-glucosidase N-acetyl-β-glucosaminidase, β-glucosidase, urease, gelatin hydrolysis and fermentation of glucose, ribose xylose, mannitol, maltose, lactose, saccharose and glycogen. Using the Api Zym (BioMerieux) system, alkaline and acid phosphatases and Naphtol-AS-BI phosphohydrolase activities are positive, but esterase (C4), esterase lipase (C8), lipase (C14), trypsin, α-chemotrypsin, α-galactosidase, β-galactosidase, β-glucuronidase, α-glucosidase, N actetyl-β-glucosaminidase, leucine arylamidase, valine arylamidase, cystin arylamidase, α-mannosidase and α-fucosidase activities are negative. Using the API 50CH system (BioMerieux), fermentation of starch, glycogen, glycerol, erythritol, esculin ferric citrate, amygdalin, arbutin, salicin, L-arabinose, D-ribose, D-xylose, methyl β-D-xylopyranoside, D-galactose, D-glucose, D-fructose, D-mannose, L-rhamnose, D-mannitol, methyl α-D-xylopyranoside, methyl α-D-glucopyranoside, N-acetylglucosamine, D-cellobiose, D-maltose, D-lactose, D-melibiose, D-saccharose, D-trehalose, inulin, D-raffinose, D-lyxose, D-arabinose, L-xylose, D-adonitol, L-sorbose, dulcitol, inositol, D-sorbitol, D-melezitose, D- xylitol, gentiobiose, D-turanose, D-tagatose, D-fucose, L-fucose, D-arabitol, L-arabitol, potassium gluconate, and potassium 2-ketogluconate are negative. Cells are susceptible to amoxicillin, ceftriaxone, imipenem, rifampicin, gentamicin, doxycycline and vancomycin but resistant to ciprofloxacin, trimethoprim/ sulfamethoxazole, eyrthromycin and metronidazole.

The G+C content of the genome is 67.2%. The 16S rRNA and genome sequences are deposited in GenBank under accession numbers HG726038 andCBYN00000000, respectively. The habitat of the microorganism is the human digestive tract. The type strain JCB^T^ (= CSUR P778 = DSM 45997) was isolated from the fecal flora of a Saudi male who suffered from morbid obesity.
